# Medication Adherence and Technology-Based Interventions for Adolescents With Chronic Health Conditions: A Few Key Considerations

**DOI:** 10.2196/mhealth.8310

**Published:** 2017-12-22

**Authors:** Sherif M Badawy, Alexis A Thompson, Lisa M Kuhns

**Affiliations:** ^1^ Division of Hematology, Oncology and Stem Cell Transplant Ann & Robert H Lurie Children's Hospital of Chicago Chicago, IL United States; ^2^ Department of Pediatrics Northwestern University Feinberg School of Medicine Chicago, IL United States; ^3^ Department of Pediatrics, Division of Hematology and Oncology Zagazig University Faculty of Medicine Zagazig Egypt; ^4^ Division of Adolescent Medicine Ann & Robert H Lurie Children's Hospital of Chicago Chicago, IL United States

**Keywords:** adolescents, adherence, compliance, technology interventions, mobile apps, text messaging

## Abstract

The number of children and adolescents with chronic health conditions (CHCs) has doubled over the past two decades. Medication adherence is a key component of disease management within these groups. Low adherence to prescribed medications is a known problem in adolescents with CHCs and is related to health outcomes, including quality of life, disease complications, and mortality. Adolescence is a critical time to create routines and health behaviors that optimize disease self-management and transition to adult care. The mounting interest in the development and use of mobile health tools provides novel opportunities to connect patients, particularly adolescents, with their providers outside of the clinic and to improve health outcomes. There is growing evidence to support the efficacy of technology-based approaches, in particular text-messaging and mobile apps, to improve adherence behavior in adolescents, although cost-effectiveness and long-term health benefits remain unclear. In this short viewpoint article, we review some important considerations for promoting medication adherence in adolescents with CHCs using technology-based approaches.

The number of children and adolescents with chronic health conditions (CHCs) continues to increase and has doubled over the past two decades, which represents an important public health concern [1]. Pediatric patients with CHCs, particularly adolescents, face challenges when trying to manage their illnesses and optimize their self-management skills. Adolescents with CHCs form a special subpopulation of pediatric patients (12-17 years old) learning how to self-manage medical decisions in preparation for an inevitable transition to adult care. Although the number of adolescents with CHCs is increasing, the number of validated self-reported scales for medication adherence that have been developed and tested specifically for adolescents is limited. Assessment of adolescent patients using parental proxy reports is not ideal. The use of tools designed for (and validated in) adults may be problematic, given the unique physiological, developmental, psychosocial, and education/vocational considerations of adolescence.

Adolescence is a critical time to create routines and health behaviors that optimize disease self-management and preparation for a seamless transition to adult care. The involvement of adolescents with CHCs in their own care can be demanding for both families and health care professionals, although it is an important investment given the short-term and long-term gains [2]. Medication adherence is a key component of disease management and low adherence to prescribed medications is a known problem in adolescents with CHCs, which is related to health outcomes, including quality of life, disease complications, and mortality [3]. Moreover, medication nonadherence has been associated with more frequent utilization of health services as well as higher health care expenses across pediatric CHCs [4]. Nevertheless, approaches to increase adherence to prescribed medications among adolescents with CHCs that are efficacious, practical, and cost-effective are lacking.

Taking daily medication(s) is a daunting task for many adolescents with CHCs, regardless of the prescribed regimen. Despite differences in disease-specific monitoring and treatment requirements among adolescents with CHCs, recent data suggest that barriers are similar across conditions [5]. Hanghoj and Boisen systematically reviewed data on perceived barriers from 2501 adolescents who had at least one of 14 chronic illnesses [5]. In order of frequency, the common barriers to medication adherence included: (1) aspects of physical well-being, such as side effects (including changes in physical appearance), reduction in symptoms/feeling well, and pill taste or swallowing problems; (2) forgetting to take medications, in part due to competing activities or changes in schedule; (3) desire to be normal and forget, ignore, or be free of their disease; and (4) lack of support from peers, parents, and health professionals. Therefore, the challenges that adolescents with CHCs need to overcome to optimize their medication adherence may be multi-faceted, but amenable to common adherence-enhancing interventions [5].

Clinics need information on evidence-based approaches to be able to implement these initiatives in the practice environment. Patient-centered and stakeholder-informed interventions developed with and for adolescents with CHCs are essential to improve adherence and enhance uptake, as well as engagement with interventions over time (particularly technology-based approaches). Access to personal technology, in particular smartphones, is becoming ubiquitous [6-8]. The mounting interest in the development and use of mobile health tools provides novel opportunities to connect patients with their providers outside of the clinic to improve health outcomes. Adolescents have adopted communication technology at a relatively fast pace, regardless of their socioeconomic status. A recent report indicates that most adolescents have widespread access to personal technology tools, including smartphones (73%), tablets (58%), desktop computers (87%), and/or laptop computers (81%) [6]. These findings suggest that technology-based interventions may present a unique opportunity to improve medication adherence and enhance self-management skills in adolescents across CHCs.

The use of personal and widely available technology-based approaches (in particular text-messaging, mobile apps, and mobile social media) to improve adherence behavior and other health outcomes in adolescents has shown overall acceptability and feasibility, with modest evidence for efficacy [9-12]. Nevertheless, the long-term health benefits, cost-effectiveness, and sustainability of patient engagement through technology-based approaches remain unclear [13,14]. Additionally, text messaging delivery methods often lack innovative features targeted to adolescents. Furthermore, methods to quantify patient fatigue, which is assumed to occur among adults with frequent text messaging, and the sustainability of patient engagement may apply differently to adolescents, representing a challenge for researchers. Therefore, while the evidence to date is encouraging and promising, further study of technology-based interventions for adolescent self-management and medication adherence, with rigorous study designs and across a wide range of CHCs, are needed. Moreover, further research is needed to explore adolescents’ insights into the role and the design of technology-based interventions in identifying facilitators or preferred strategies to improve medication adherence. The consistent use of reporting guidelines for technology-based interventions is also critical to support the evidence generated, and conclusions that can be drawn, from adherence intervention studies [15].

While research efforts continue to produce better evidence for these technologies to promote health outcomes among adolescents with CHCs, we encourage medical providers to begin a conversation with leadership within their provider group or hospital about the incorporation of mobile technology into the practice environment, and to ask patients about their use of mobile technology and apps to promote self-care.

In conclusion, the number of adolescents with chronic illnesses continues to increase. Medication nonadherence is a challenge in adolescents across chronic conditions. Adolescents are frequent users of technology and engaging adolescents with chronic illnesses in their self-management could be invaluable for improving long-term outcomes. The use of technology-based interventions to improve medication adherence has shown promising results, and seeking adolescents’ perspectives could enhance uptake and long-term engagement, and minimize patient fatigue. Following guidelines for reporting results of technology-based interventions, and validating adolescent-specific adherence assessment instruments, would enhance further comparative research across studies.

## Abbreviations

CHC: chronic health condition
